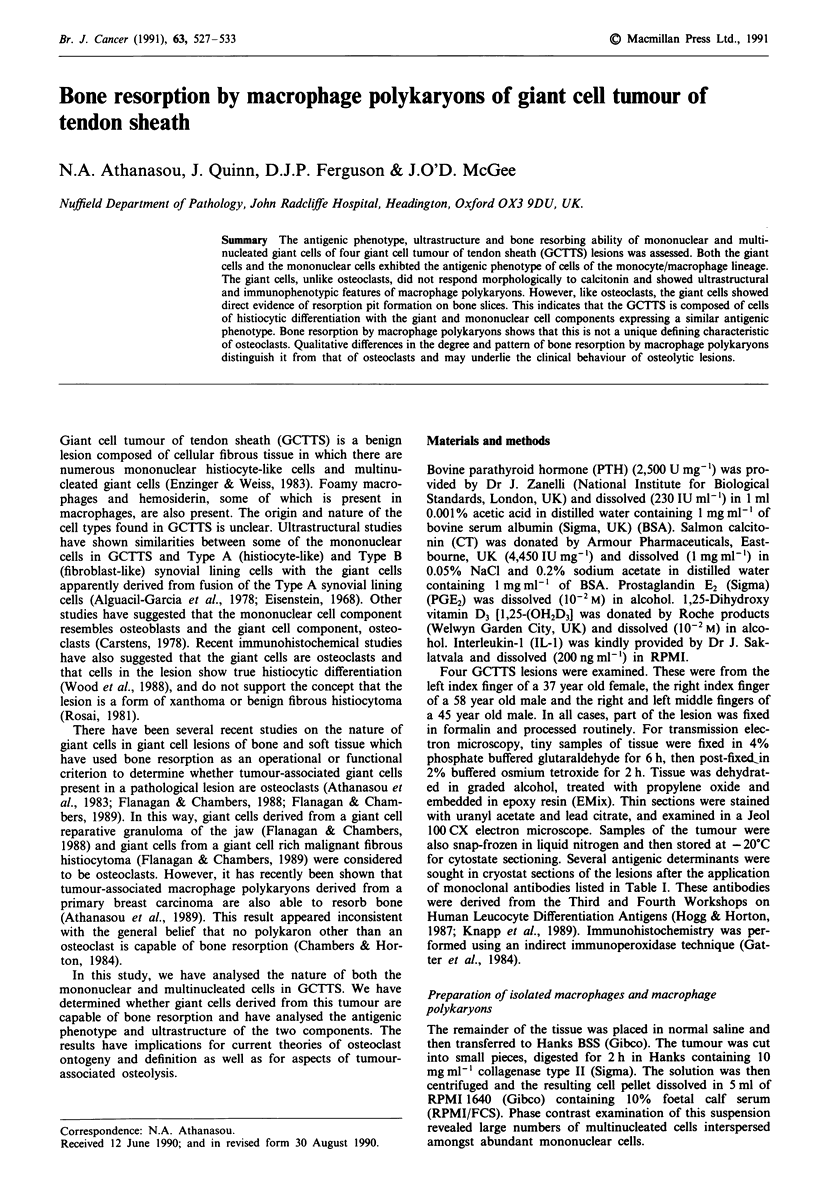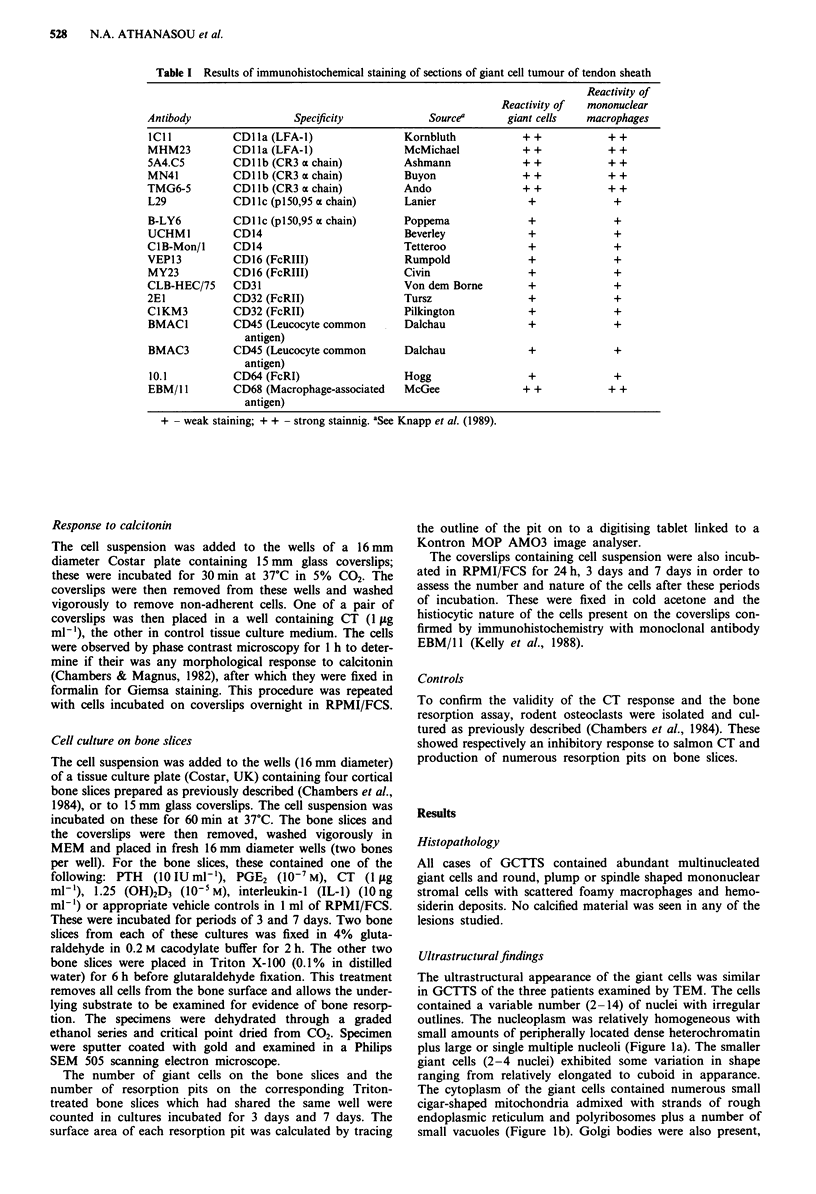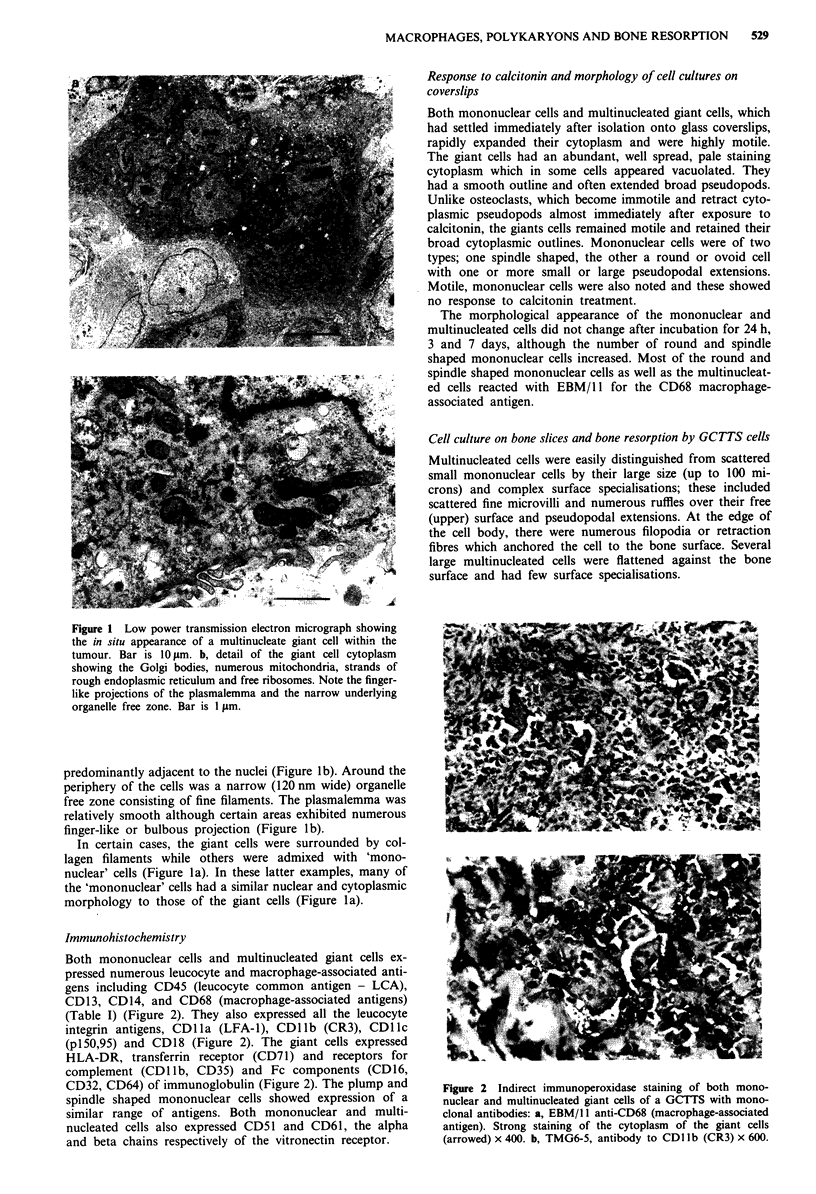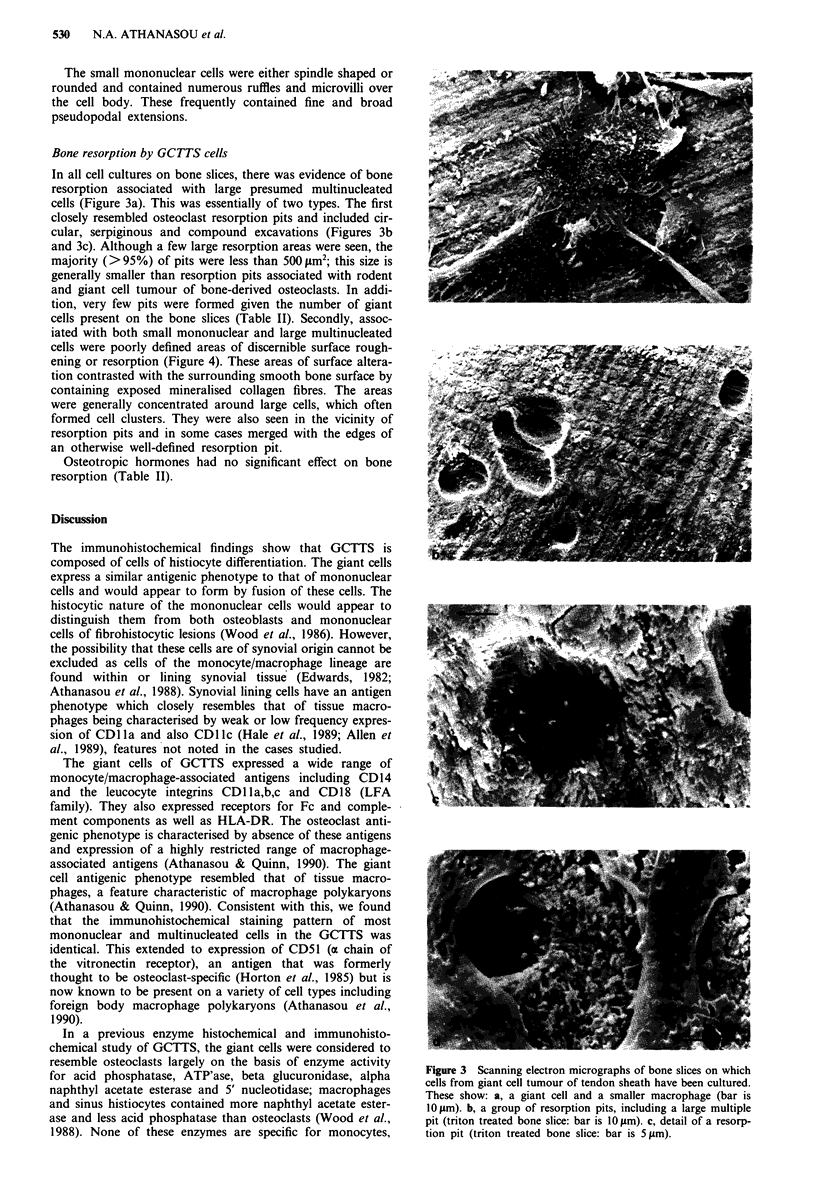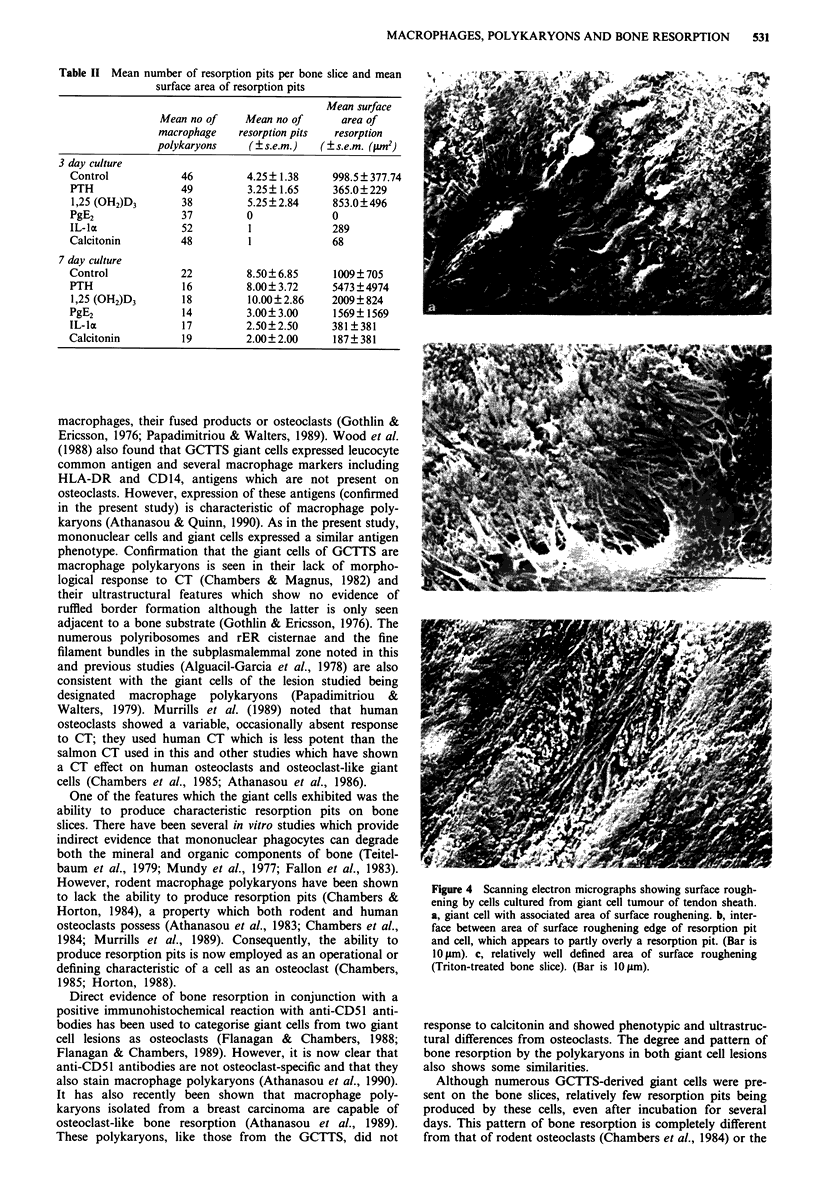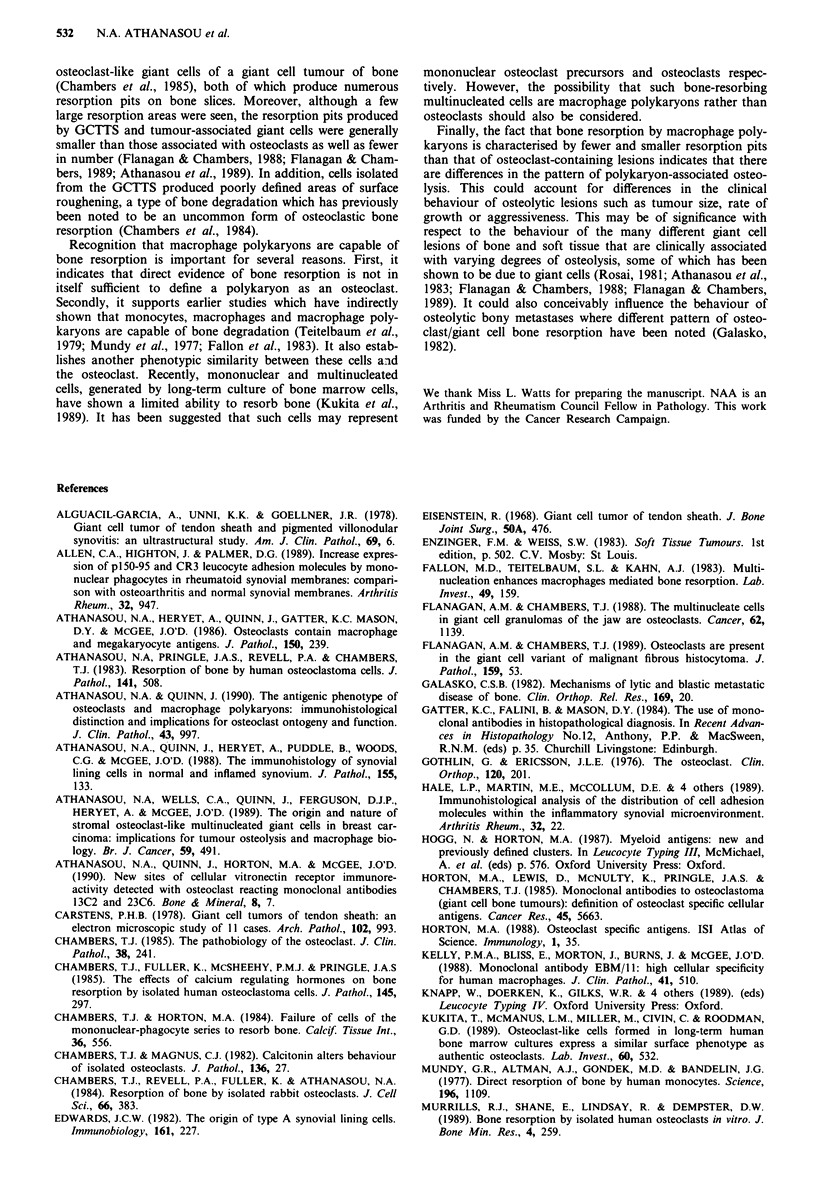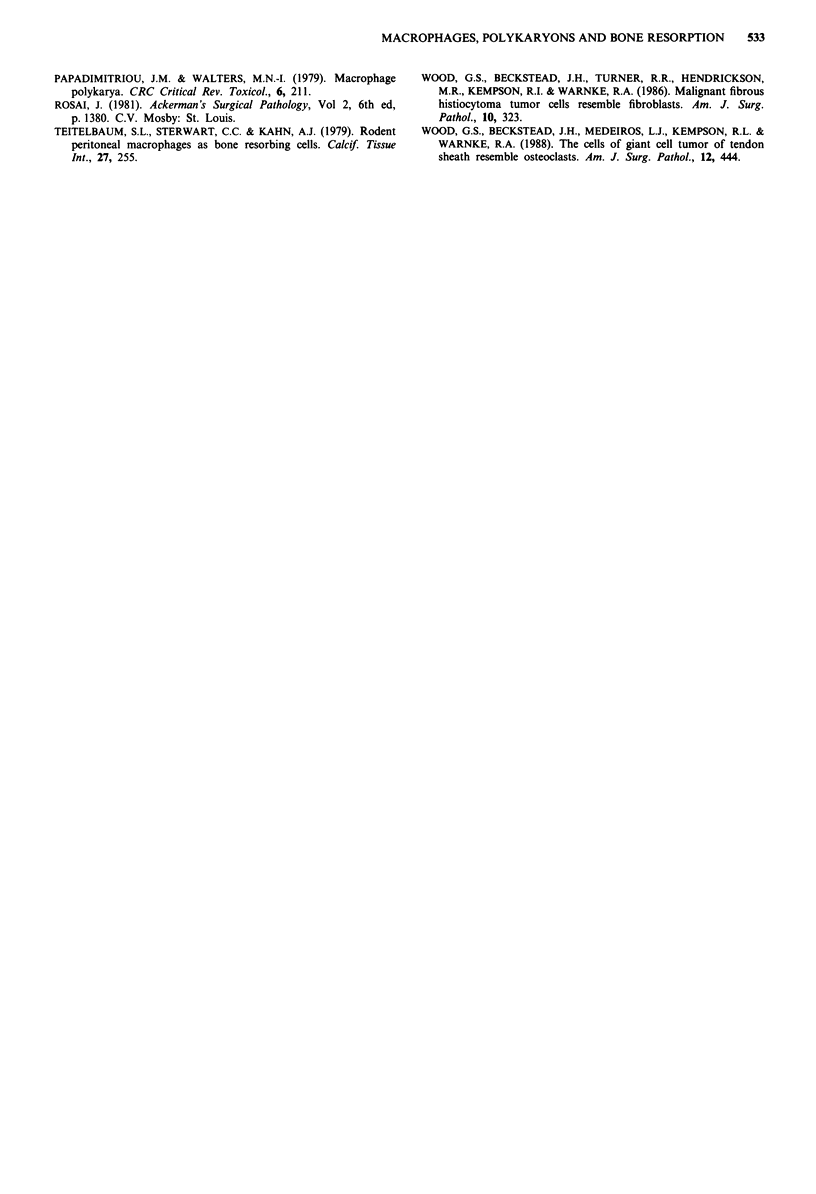# Bone resorption by macrophage polykaryons of giant cell tumour of tendon sheath.

**DOI:** 10.1038/bjc.1991.125

**Published:** 1991-04

**Authors:** N. A. Athanasou, J. Quinn, D. J. Ferguson, J. O. McGee

**Affiliations:** Nuffield Department of Pathology, John Radcliffe Hospital, Headington, Oxford, UK.

## Abstract

**Images:**


Br. J. Cancer (1991), 63, 527-533 © Macmillan Press Ltd., 1991

### Bone resorption by macrophage polykaryons of giant cell tumour of tendon sheath

N.A. Athanasou, J. Quinn, D.J.P. Ferguson & J.O'D. McGee

*Nuffield Department of Pathology, John Radcliffe Hospital, Headington, Oxford OX3 9DU, UK.*

**Summary** The antigenic phenotype, ultrastructure and bone resorbing ability of mononuclear and multinucleated giant cells of four giant cell tumour of tendon sheath (GCTTS) lesions was assessed. Both the giant cells and the mononuclear cells exhibted the antigenic phenotype of cells of the monocyte/macrophage lineage. The giant cells, unlike osteoclasts, did not respond morphologically to calcitonin and showed ultrastructural and immunophenotypic features of macrophage polykaryons. However, like osteoclasts, the giant cells showed direct evidence of resorption pit formation on bone slices. This indicates that the GCTTS is composed of cells of histiocytic differentiation with the giant and mononuclear cell components expressing a similar antigenic phenotype. Bone resorption by macrophage polykaryons shows that this is not a unique defining characteristic of osteoclasts. Qualitative differences in the degree and pattern of bone resorption by macrophage polykaryons distinguish it from that of osteoclasts and may underlie the clinical behaviour of osteolytic lesions.

Giant cell tumour of tendon sheath (GCTTS) is a benign lesion composed of cellular fibrous tissue in which there are numerous mononuclear histiocyte-like cells and multinucleated giant cells (Enzinger & Weiss, 1983). Foamy macrophages and hemosiderin, some of which is present in macrophages, are also present. The origin and nature of the cell types found in GCTTS is unclear. Ultrastructural studies have shown similarities between some of the mononuclear cells in GCTTS and Type A (histiocyte-like) and Type B (fibroblast-like) synovial lining cells with the giant cells apparently derived from fusion of the Type A synovial lining cells (Alguacil-Garcia *et al.*, 1978; Eisenstein, 1968). Other studies have suggested that the mononuclear cell component resembles osteoblasts and the giant cell component, osteoclasts (Carstens, 1978). Recent immunohistochemical studies have also suggested that the giant cells are osteoclasts and that cells in the lesion show true histiocytic differentiation (Wood *et al.*, 1988), and do not support the concept that the lesion is a form of xanthoma or benign fibrous histiocytoma (Rosai, 1981).

There have been several recent studies on the nature of giant cells in giant cell lesions of bone and soft tissue which have used bone resorption as an operational or functional criterion to determine whether tumour-associated giant cells present in a pathological lesion are osteoclasts (Athanasou *et al.*, 1983; Flanagan & Chambers, 1988; Flanagan & Chambers, 1989). In this way, giant cells derived from a giant cell reparative granuloma of the jaw (Flanagan & Chambers, 1988) and giant cells from a giant cell rich malignant fibrous histiocytoma (Flanagan & Chambers, 1989) were considered to be osteoclasts. However, it has recently been shown that tumour-associated macrophage polykaryons derived from a primary breast carcinoma are also able to resorb bone (Athanasou *et al.*, 1989). This result appeared inconsistent with the general belief that no polykaron other than an osteoclast is capable of bone resorption (Chambers & Horton, 1984).

In this study, we have analysed the nature of both the mononuclear and multinucleated cells in GCTTS. We have determined whether giant cells derived from this tumour are capable of bone resorption and have analysed the antigenic phenotype and ultrastructure of the two components. The results have implications for current theories of osteoclast ontogeny and definition as well as for aspects of tumour-associated osteolysis.

Correspondence: N.A. Athanasou.
Received 12 June 1990; and in revised form 30 August 1990.

### Materials and methods

Bovine parathyroid hormone (PTH) (2,500 U $mg^{-1}$) was provided by Dr J. Zanelli (National Institute for Biological Standards, London, UK) and dissolved (230 IU $ml^{-1}$) in 1 ml 0.001% acetic acid in distilled water containing 1 mg $ml^{-1}$ of bovine serum albumin (Sigma, UK) (BSA). Salmon calcitonin (CT) was donated by Armour Pharmaceuticals, Eastbourne, UK (4,450 IU $mg^{-1}$) and dissolved (1 mg $ml^{-1}$) in 0.05% NaCl and 0.2% sodium acetate in distilled water containing 1 mg $ml^{-1}$ of BSA. Prostaglandin $E_2$ (Sigma) ($PGE_2$) was dissolved ($10^{-2}$ M) in alcohol. 1,25-Dihydroxy vitamin $D_3$ [1,25-($OH_2D_3$] was donated by Roche products (Welwyn Garden City, UK) and dissolved ($10^{-2}$ M) in alcohol. Interleukin-1 (IL-1) was kindly provided by Dr J. Saklatvala and dissolved (200 ng $ml^{-1}$) in RPMI.

Four GCTTS lesions were examined. These were from the left index finger of a 37 year old female, the right index finger of a 58 year old male and the right and left middle fingers of a 45 year old male. In all cases, part of the lesion was fixed in formalin and processed routinely. For transmission electron microscopy, tiny samples of tissue were fixed in 4% phosphate buffered glutaraldehyde for 6 h, then post-fixed in 2% buffered osmium tetroxide for 2 h. Tissue was dehydrated in graded alcohol, treated with propylene oxide and embedded in epoxy resin (EMix). Thin sections were stained with uranyl acetate and lead citrate, and examined in a Jeol 100 CX electron microscope. Samples of the tumour were also snap-frozen in liquid nitrogen and then stored at $-20°C$ for cytostate sectioning. Several antigenic determinants were sought in cryostat sections of the lesions after the application of monoclonal antibodies listed in Table I. These antibodies were derived from the Third and Fourth Workshops on Human Leucocyte Differentiation Antigens (Hogg & Horton, 1987; Knapp *et al.*, 1989). Immunohistochemistry was performed using an indirect immunoperoxidase technique (Gatter *et al.*, 1984).

### *Preparation of isolated macrophages and macrophage polykaryons*

The remainder of the tissue was placed in normal saline and then transferred to Hanks BSS (Gibco). The tumour was cut into small pieces, digested for 2 h in Hanks containing 10 mg $ml^{-1}$ collagenase type II (Sigma). The solution was then centrifuged and the resulting cell pellet dissolved in 5 ml of RPMI 1640 (Gibco) containing 10% foetal calf serum (RPMI/FCS). Phase contrast examination of this suspension revealed large numbers of multinucleated cells interspersed amongst abundant mononuclear cells.

**Table I** Results of immunohistochemical staining of sections of giant cell tumour of tendon sheath

| *Antibody* | *Specificity* | *Source*[a] | *Reactivity of giant cells* | *Reactivity of mononuclear macrophages* |
|---|---|---|---|---|
| 1C11 | CD11a (LFA-1) | Kornbluth | ++ | ++ |
| MHM23 | CD11a (LFA-1) | McMichael | ++ | ++ |
| 5A4.C5 | CD11b (CR3 α chain) | Ashmann | ++ | ++ |
| MN41 | CD11b (CR3 α chain) | Buyon | ++ | ++ |
| TMG6-5 | CD11b (CR3 α chain) | Ando | ++ | ++ |
| L29 | CD11c (p150,95 α chain) | Lanier | + | + |
| B-LY6 | CD11c (p150,95 α chain) | Poppema | + | + |
| UCHM1 | CD14 | Beverley | + | + |
| C1B-Mon/1 | CD14 | Tetteroo | + | + |
| VEP13 | CD16 (FcRIII) | Rumpold | + | + |
| MY23 | CD16 (FcRIII) | Civin | + | + |
| CLB-HEC/75 | CD31 | Von dem Borne | + | + |
| 2E1 | CD32 (FcRII) | Tursz | + | + |
| C1KM3 | CD32 (FcRII) | Pilkington | + | + |
| BMAC1 | CD45 (Leucocyte common antigen) | Dalchau | + | + |
| BMAC3 | CD45 (Leucocyte common antigen) | Dalchau | + | + |
| 10.1 | CD64 (FcRI) | Hogg | + | + |
| EBM/11 | CD68 (Macrophage-associated antigen) | McGee | ++ | ++ |

+ – weak staining; ++ – strong stainnig. [a]See Knapp *et al.* (1989).

*Response to calcitonin*

The cell suspension was added to the wells of a 16 mm diameter Costar plate containing 15 mm glass coverslips; these were incubated for 30 min at 37°C in 5% $CO_2$. The coverslips were then removed from these wells and washed vigorously to remove non-adherent cells. One of a pair of coverslips was then placed in a well containing CT (1 μg $ml^{-1}$), the other in control tissue culture medium. The cells were observed by phase contrast microscopy for 1 h to determine if their was any morphological response to calcitonin (Chambers & Magnus, 1982), after which they were fixed in formalin for Giemsa staining. This procedure was repeated with cells incubated on coverslips overnight in RPMI/FCS.

*Cell culture on bone slices*

The cell suspension was added to the wells (16 mm diameter) of a tissue culture plate (Costar, UK) containing four cortical bone slices prepared as previously described (Chambers *et al.*, 1984), or to 15 mm glass coverslips. The cell suspension was incubated on these for 60 min at 37°C. The bone slices and the coverslips were then removed, washed vigorously in MEM and placed in fresh 16 mm diameter wells (two bones per well). For the bone slices, these contained one of the following: PTH (10 IU $ml^{-1}$), $PGE_2$ ($10^{-7}$ M), CT (1 μg $ml^{-1}$), 1.25 $(OH)_2D_3$ ($10^{-5}$ M), interleukin-1 (IL-1) (10 ng $ml^{-1}$) or appropriate vehicle controls in 1 ml of RPMI/FCS. These were incubated for periods of 3 and 7 days. Two bone slices from each of these cultures was fixed in 4% glutaraldehyde in 0.2 M cacodylate buffer for 2 h. The other two bone slices were placed in Triton X-100 (0.1% in distilled water) for 6 h before glutaraldehyde fixation. This treatment removes all cells from the bone surface and allows the underlying substrate to be examined for evidence of bone resorption. The specimens were dehydrated through a graded ethanol series and critical point dried from $CO_2$. Specimen were sputter coated with gold and examined in a Philips SEM 505 scanning electron microscope.

The number of giant cells on the bone slices and the number of resorption pits on the corresponding Triton-treated bone slices which had shared the same well were counted in cultures incubated for 3 days and 7 days. The surface area of each resorption pit was calculated by tracing the outline of the pit on to a digitising tablet linked to a Kontron MOP AMO3 image analyser.

The coverslips containing cell suspension were also incubated in RPMI/FCS for 24 h, 3 days and 7 days in order to assess the number and nature of the cells after these periods of incubation. These were fixed in cold acetone and the histiocytic nature of the cells present on the coverslips confirmed by immunohistochemistry with monoclonal antibody EBM/11 (Kelly *et al.*, 1988).

*Controls*

To confirm the validity of the CT response and the bone resorption assay, rodent osteoclasts were isolated and cultured as previously described (Chambers *et al.*, 1984). These showed respectively an inhibitory response to salmon CT and production of numerous resorption pits on bone slices.

### Results

*Histopathology*

All cases of GCTTS contained abundant multinucleated giant cells and round, plump or spindle shaped mononuclear stromal cells with scattered foamy macrophages and hemosiderin deposits. No calcified material was seen in any of the lesions studied.

*Ultrastructural findings*

The ultrastructural appearance of the giant cells was similar in GCTTS of the three patients examined by TEM. The cells contained a variable number (2–14) of nuclei with irregular outlines. The nucleoplasm was relatively homogeneous with small amounts of peripherally located dense heterochromatin plus large or single multiple nucleoli (Figure 1a). The smaller giant cells (2–4 nuclei) exhibited some variation in shape ranging from relatively elongated to cuboid in apparance. The cytoplasm of the giant cells contained numerous small cigar-shaped mitochondria admixed with strands of rough endoplasmic reticulum and polyribosomes plus a number of small vacuoles (Figure 1b). Golgi bodies were also present,

**Figure 1** Low power transmission electron micrograph showing the *in situ* appearance of a multinucleate giant cell within the tumour. Bar is 10 μm. **b**, detail of the giant cell cytoplasm showing the Golgi bodies, numerous mitochondria, strands of rough endoplasmic reticulum and free ribosomes. Note the finger-like projections of the plasmalemma and the narrow underlying organelle free zone. Bar is 1 μm.

predominantly adjacent to the nuclei (Figure 1b). Around the periphery of the cells was a narrow (120 nm wide) organelle free zone consisting of fine filaments. The plasmalemma was relatively smooth although certain areas exhibited numerous finger-like or bulbous projection (Figure 1b).

In certain cases, the giant cells were surrounded by collagen filaments while others were admixed with 'mononuclear' cells (Figure 1a). In these latter examples, many of the 'mononuclear' cells had a similar nuclear and cytoplasmic morphology to those of the giant cells (Figure 1a).

*Immunohistochemistry*

Both mononuclear cells and multinucleated giant cells expressed numerous leucocyte and macrophage-associated antigens including CD45 (leucocyte common antigen – LCA), CD13, CD14, and CD68 (macrophage-associated antigens) (Table I) (Figure 2). They also expressed all the leucocyte integrin antigens, CD11a (LFA-1), CD11b (CR3), CD11c (p150,95) and CD18 (Figure 2). The giant cells expressed HLA-DR, transferrin receptor (CD71) and receptors for complement (CD11b, CD35) and Fc components (CD16, CD32, CD64) of immunoglobulin (Figure 2). The plump and spindle shaped mononuclear cells showed expression of a similar range of antigens. Both mononuclear and multinucleated cells also expressed CD51 and CD61, the alpha and beta chains respectively of the vitronectin receptor.

*Response to calcitonin and morphology of cell cultures on coverslips*

Both mononuclear cells and multinucleated giant cells, which had settled immediately after isolation onto glass coverslips, rapidly expanded their cytoplasm and were highly motile. The giant cells had an abundant, well spread, pale staining cytoplasm which in some cells appeared vacuolated. They had a smooth outline and often extended broad pseudopods. Unlike osteoclasts, which become immotile and retract cytoplasmic pseudopods almost immediately after exposure to calcitonin, the giants cells remained motile and retained their broad cytoplasmic outlines. Mononuclear cells were of two types; one spindle shaped, the other a round or ovoid cell with one or more small or large pseudopodal extensions. Motile, mononuclear cells were also noted and these showed no response to calcitonin treatment.

The morphological appearance of the mononuclear and multinucleated cells did not change after incubation for 24 h, 3 and 7 days, although the number of round and spindle shaped mononuclear cells increased. Most of the round and spindle shaped mononuclear cells as well as the multinucleated cells reacted with EBM/11 for the CD68 macrophage-associated antigen.

*Cell culture on bone slices and bone resorption by GCTTS cells*

Multinucleated cells were easily distinguished from scattered small mononuclear cells by their large size (up to 100 microns) and complex surface specialisations; these included scattered fine microvilli and numerous ruffles over their free (upper) surface and pseudopodal extensions. At the edge of the cell body, there were numerous filopodia or retraction fibres which anchored the cell to the bone surface. Several large multinucleated cells were flattened against the bone surface and had few surface specialisations.

**Figure 2** Indirect immunoperoxidase staining of both mononuclear and multinucleated giant cells of a GCTTS with monoclonal antibodies: **a**, EBM/11 anti-CD68 (macrophage-associated antigen). Strong staining of the cytoplasm of the giant cells (arrowed) × 400. **b**, TMG6-5, antibody to CD11b (CR3) × 600.

The small mononuclear cells were either spindle shaped or rounded and contained numerous ruffles and microvilli over the cell body. These frequently contained fine and broad pseudopodal extensions.

*Bone resorption by GCTTS cells*

In all cell cultures on bone slices, there was evidence of bone resorption associated with large presumed multinucleated cells (Figure 3a). This was essentially of two types. The first closely resembled osteoclast resorption pits and included circular, serpiginous and compound excavations (Figures 3b and 3c). Although a few large resorption areas were seen, the majority (>95%) of pits were less than 500 $\mu m^2$; this size is generally smaller than resorption pits associated with rodent and giant cell tumour of bone-derived osteoclasts. In addition, very few pits were formed given the number of giant cells present on the bone slices (Table II). Secondly, associated with both small mononuclear and large multinucleated cells were poorly defined areas of discernible surface roughening or resorption (Figure 4). These areas of surface alteration contrasted with the surrounding smooth bone surface by containing exposed mineralised collagen fibres. The areas were generally concentrated around large cells, which often formed cell clusters. They were also seen in the vicinity of resorption pits and in some cases merged with the edges of an otherwise well-defined resorption pit.

Osteotropic hormones had no significant effect on bone resorption (Table II).

### Discussion

The immunohistochemical findings show that GCTTS is composed of cells of histiocyte differentiation. The giant cells express a similar antigenic phenotype to that of mononuclear cells and would appear to form by fusion of these cells. The histocytic nature of the mononuclear cells would appear to distinguish them from both osteoblasts and mononuclear cells of fibrohistocytic lesions (Wood *et al*., 1986). However, the possibility that these cells are of synovial origin cannot be excluded as cells of the monocyte/macrophage lineage are found within or lining synovial tissue (Edwards, 1982; Athanasou *et al*., 1988). Synovial lining cells have an antigen phenotype which closely resembles that of tissue macrophages being characterised by weak or low frequency expression of CD11a and also CD11c (Hale *et al*., 1989; Allen *et al*., 1989), features not noted in the cases studied.

The giant cells of GCTTS expressed a wide range of monocyte/macrophage-associated antigens including CD14 and the leucocyte integrins CD11a,b,c and CD18 (LFA family). They also expressed receptors for Fc and complement components as well as HLA-DR. The osteoclast antigenic phenotype is characterised by absence of these antigens and expression of a highly restricted range of macrophage-associated antigens (Athanasou & Quinn, 1990). The giant cell antigenic phenotype resembled that of tissue macrophages, a feature characteristic of macrophage polykaryons (Athanasou & Quinn, 1990). Consistent with this, we found that the immunohistochemical staining pattern of most mononuclear and multinucleated cells in the GCTTS was identical. This extended to expression of CD51 (α chain of the vitronectin receptor), an antigen that was formerly thought to be osteoclast-specific (Horton *et al*., 1985) but is now known to be present on a variety of cell types including foreign body macrophage polykaryons (Athanasou *et al*., 1990).

In a previous enzyme histochemical and immunohistochemical study of GCTTS, the giant cells were considered to resemble osteoclasts largely on the basis of enzyme activity for acid phosphatase, ATP'ase, beta glucuronidase, alpha naphthyl acetate esterase and 5′ nucleotidase; macrophages and sinus histiocytes contained more naphthyl acetate esterase and less acid phosphatase than osteoclasts (Wood *et al*., 1988). None of these enzymes are specific for monocytes,

**Figure 3** Scanning electron micrographs of bone slices on which cells from giant cell tumour of tendon sheath have been cultured. These show: **a**, a giant cell and a smaller macrophage (bar is 10 μm). **b**, a group of resorption pits, including a large multiple pit (triton treated bone slice: bar is 10 μm). **c**, detail of a resorption pit (triton treated bone slice: bar is 5 μm).

**Table II** Mean number of resorption pits per bone slice and mean surface area of resorption pits

| | *Mean no of macrophage polykaryons* | *Mean no of resorption pits (±s.e.m.)* | *Mean surface area of resorption (±s.e.m. (μm²)* |
|---|---|---|---|
| *3 day culture* | | | |
| Control | 46 | 4.25±1.38 | 998.5±377.74 |
| PTH | 49 | 3.25±1.65 | 365.0±229 |
| 1,25 $(OH_2)D_3$ | 38 | 5.25±2.84 | 853.0±496 |
| $PgE_2$ | 37 | 0 | 0 |
| IL-1α | 52 | 1 | 289 |
| Calcitonin | 48 | 1 | 68 |
| *7 day culture* | | | |
| Control | 22 | 8.50±6.85 | 1009±705 |
| PTH | 16 | 8.00±3.72 | 5473±4974 |
| 1,25 $(OH_2)D_3$ | 18 | 10.00±2.86 | 2009±824 |
| $PgE_2$ | 14 | 3.00±3.00 | 1569±1569 |
| IL-1α | 17 | 2.50±2.50 | 381±381 |
| Calcitonin | 19 | 2.00±2.00 | 187±381 |

macrophages, their fused products or osteoclasts (Gothlin & Ericsson, 1976; Papadimitriou & Walters, 1989). Wood *et al.* (1988) also found that GCTTS giant cells expressed leucocyte common antigen and several macrophage markers including HLA-DR and CD14, antigens which are not present on osteoclasts. However, expression of these antigens (confirmed in the present study) is characteristic of macrophage polykaryons (Athanasou & Quinn, 1990). As in the present study, mononuclear cells and giant cells expressed a similar antigen phenotype. Confirmation that the giant cells of GCTTS are macrophage polykaryons is seen in their lack of morphological response to CT (Chambers & Magnus, 1982) and their ultrastructural features which show no evidence of ruffled border formation although the latter is only seen adjacent to a bone substrate (Gothlin & Ericsson, 1976). The numerous polyribosomes and rER cisternae and the fine filament bundles in the subplasmalemmal zone noted in this and previous studies (Alguacil-Garcia *et al.*, 1978) are also consistent with the giant cells of the lesion studied being designated macrophage polykaryons (Papadimitriou & Walters, 1979). Murrills *et al.* (1989) noted that human osteoclasts showed a variable, occasionally absent response to CT; they used human CT which is less potent than the salmon CT used in this and other studies which have shown a CT effect on human osteoclasts and osteoclast-like giant cells (Chambers *et al.*, 1985; Athanasou *et al.*, 1986).

One of the features which the giant cells exhibited was the ability to produce characteristic resorption pits on bone slices. There have been several *in vitro* studies which provide indirect evidence that mononuclear phagocytes can degrade both the mineral and organic components of bone (Teitelbaum *et al.*, 1979; Mundy *et al.*, 1977; Fallon *et al.*, 1983). However, rodent macrophage polykaryons have been shown to lack the ability to produce resorption pits (Chambers & Horton, 1984), a property which both rodent and human osteoclasts possess (Athanasou *et al.*, 1983; Chambers *et al.*, 1984; Murrills *et al.*, 1989). Consequently, the ability to produce resorption pits is now employed as an operational or defining characteristic of a cell as an osteoclast (Chambers, 1985; Horton, 1988).

Direct evidence of bone resorption in conjunction with a positive immunohistochemical reaction with anti-CD51 antibodies has been used to categorise giant cells from two giant cell lesions as osteoclasts (Flanagan & Chambers, 1988; Flanagan & Chambers, 1989). However, it is now clear that anti-CD51 antibodies are not osteoclast-specific and that they also stain macrophage polykaryons (Athanasou *et al.*, 1990). It has also recently been shown that macrophage polykaryons isolated from a breast carcinoma are capable of osteoclast-like bone resorption (Athanasou *et al.*, 1989). These polykaryons, like those from the GCTTS, did not response to calcitonin and showed phenotypic and ultrastructural differences from osteoclasts. The degree and pattern of bone resorption by the polykaryons in both giant cell lesions also shows some similarities.

**Figure 4** Scanning electron micrographs showing surface roughening by cells cultured from giant cell tumour of tendon sheath. **a**, giant cell with associated area of surface roughening. **b**, interface between area of surface roughening edge of resorption pit and cell, which appears to partly overly a resorption pit. (Bar is 10 μm). **c**, relatively well defined area of surface roughening (Triton-treated bone slice). (Bar is 10 μm).

Although numerous GCTTS-derived giant cells were present on the bone slices, relatively few resorption pits being produced by these cells, even after incubation for several days. This pattern of bone resorption is completely different from that of rodent osteoclasts (Chambers *et al.*, 1984) or the osteoclast-like giant cells of a giant cell tumour of bone (Chambers *et al.*, 1985), both of which produce numerous resorption pits on bone slices. Moreover, although a few large resorption areas were seen, the resorption pits produced by GCTTS and tumour-associated giant cells were generally smaller than those associated with osteoclasts as well as fewer in number (Flanagan & Chambers, 1988; Flanagan & Chambers, 1989; Athanasou *et al.*, 1989). In addition, cells isolated from the GCTTS produced poorly defined areas of surface roughening, a type of bone degradation which has previously been noted to be an uncommon form of osteoclastic bone resorption (Chambers *et al.*, 1984).

Recognition that macrophage polykaryons are capable of bone resorption is important for several reasons. First, it indicates that direct evidence of bone resorption is not in itself sufficient to define a polykaryon as an osteoclast. Secondly, it supports earlier studies which have indirectly shown that monocytes, macrophages and macrophage polykaryons are capable of bone degradation (Teitelbaum *et al.*, 1979; Mundy *et al.*, 1977; Fallon *et al.*, 1983). It also establishes another phenotypic similarity between these cells and the osteoclast. Recently, mononuclear and multinucleated cells, generated by long-term culture of bone marrow cells, have shown a limited ability to resorb bone (Kukita *et al.*, 1989). It has been suggested that such cells may represent mononuclear osteoclast precursors and osteoclasts respectively. However, the possibility that such bone-resorbing multinucleated cells are macrophage polykaryons rather than osteoclasts should also be considered.

Finally, the fact that bone resorption by macrophage polykaryons is characterised by fewer and smaller resorption pits than that of osteoclast-containing lesions indicates that there are differences in the pattern of polykaryon-associated osteolysis. This could account for differences in the clinical behaviour of osteolytic lesions such as tumour size, rate of growth or aggressiveness. This may be of significance with respect to the behaviour of the many different giant cell lesions of bone and soft tissue that are clinically associated with varying degrees of osteolysis, some of which has been shown to be due to giant cells (Rosai, 1981; Athanasou *et al.*, 1983; Flanagan & Chambers, 1988; Flanagan & Chambers, 1989). It could also conceivably influence the behaviour of osteolytic bony metastases where different pattern of osteoclast/giant cell bone resorption have been noted (Galasko, 1982).

We thank Miss L. Watts for preparing the manuscript. NAA is an Arthritis and Rheumatism Council Fellow in Pathology. This work was funded by the Cancer Research Campaign.

### References

ALGUACIL-GARCIA, A., UNNI, K.K. & GOELLNER, J.R. (1978). Giant cell tumor of tendon sheath and pigmented villonodular synovitis: an ultrastructural study. *Am. J. Clin. Pathol.*, **69**, 6.

ALLEN, C.A., HIGHTON, J. & PALMER, D.G. (1989). Increase expression of p150-95 and CR3 leucocyte adhesion molecules by mononuclear phagocytes in rheumatoid synovial membranes: comparison with osteoarthritis and normal synovial membranes. *Arthritis Rheum.*, **32**, 947.

ATHANASOU, N.A., HERYET, A., QUINN, J., GATTER, K.C. MASON, D.Y. & MCGEE, J.O'D. (1986). Osteoclasts contain macrophage and megakaryocyte antigens. *J. Pathol.*, **150**, 239.

ATHANASOU, N.A, PRINGLE, J.A.S., REVELL, P.A. & CHAMBERS, T.J. (1983). Resorption of bone by human osteoclastoma cells. *J. Pathol.*, **141**, 508.

ATHANASOU, N.A. & QUINN, J. (1990). The antigenic phenotype of osteoclasts and macrophage polykaryons: immunohistological distinction and implications for osteoclast ontogeny and function. *J. Clin. Pathol.*, **43**, 997.

ATHANASOU, N.A., QUINN, J., HERYET, A., PUDDLE, B., WOODS, C.G. & MCGEE, J.O'D. (1988). The immunohistology of synovial lining cells in normal and inflamed synovium. *J. Pathol.*, **155**, 133.

ATHANASOU, N.A, WELLS, C.A., QUINN, J., FERGUSON, D.J.P., HERYET, A. & MCGEE, J.O'D. (1989). The origin and nature of stromal osteoclast-like multinucleated giant cells in breast carcinoma: implications for tumour osteolysis and macrophage biology. *Br. J. Cancer*, **59**, 491.

ATHANASOU, N.A., QUINN, J., HORTON, M.A. & MCGEE, J.O'D. (1990). New sites of cellular vitronectin receptor immunoreactivity detected with osteoclast reacting monoclonal antibodies 13C2 and 23C6. *Bone & Mineral*, **8**, 7.

CARSTENS, P.H.B. (1978). Giant cell tumors of tendon sheath: an electron microscopic study of 11 cases. *Arch. Pathol.*, **102**, 993.

CHAMBERS, T.J. (1985). The pathobiology of the osteoclast. *J. Clin. Pathol.*, **38**, 241.

CHAMBERS, T.J., FULLER, K., MCSHEEHY, P.M.J. & PRINGLE, J.A.S (1985). The effects of calcium regulating hormones on bone resorption by isolated human osteoclastoma cells. *J. Pathol.*, **145**, 297.

CHAMBERS, T.J. & HORTON, M.A. (1984). Failure of cells of the mononuclear-phagocyte series to resorb bone. *Calcif. Tissue Int.*, **36**, 556.

CHAMBERS, T.J. & MAGNUS, C.J. (1982). Calcitonin alters behaviour of isolated osteoclasts. *J. Pathol.*, **136**, 27.

CHAMBERS, T.J., REVELL, P.A., FULLER, K. & ATHANASOU, N.A. (1984). Resorption of bone by isolated rabbit osteoclasts. *J. Cell Sci.*, **66**, 383.

EDWARDS, J.C.W. (1982). The origin of type A synovial lining cells. *Immunobiology*, **161**, 227.

EISENSTEIN, R. (1968). Giant cell tumor of tendon sheath. *J. Bone Joint Surg.*, **50A**, 476.

ENZINGER, F.M. & WEISS, S.W. (1983). *Soft Tissue Tumours*. 1st edition, p. 502. C.V. Mosby: St Louis.

FALLON, M.D., TEITELBAUM, S.L. & KAHN, A.J. (1983). Multinucleation enhances macrophages mediated bone resorption. *Lab. Invest.*, **49**, 159.

FLANAGAN, A.M. & CHAMBERS, T.J. (1988). The multinucleate cells in giant cell granulomas of the jaw are osteoclasts. *Cancer*, **62**, 1139.

FLANAGAN, A.M. & CHAMBERS, T.J. (1989). Osteoclasts are present in the giant cell variant of malignant fibrous histocytoma. *J. Pathol.*, **159**, 53.

GALASKO, C.S.B. (1982). Mechanisms of lytic and blastic metastatic disease of bone. *Clin. Orthop. Rel. Res.*, **169**, 20.

GATTER, K.C., FALINI, B. & MASON, D.Y. (1984). The use of monoclonal antibodies in histopathological diagnosis. In *Recent Advances in Histopathology* No.12, Anthony, P.P. & MacSween, R.N.M. (eds) p. 35. Churchill Livingstone: Edinburgh.

GOTHLIN, G. & ERICSSON, J.L.E. (1976). The osteoclast. *Clin. Orthop.*, **120**, 201.

HALE, L.P., MARTIN, M.E., MCCOLLUM, D.E. & 4 others (1989). Immunohistological analysis of the distribution of cell adhesion molecules within the inflammatory synovial microenvironment. *Arthritis Rheum.*, **32**, 22.

HOGG, N. & HORTON, M.A. (1987). Myeloid antigens: new and previously defined clusters. In *Leucocyte Typing III*, McMichael, A. *et al.* (eds) p. 576. Oxford University Press: Oxford.

HORTON, M.A., LEWIS, D., MCNULTY, K., PRINGLE, J.A.S. & CHAMBERS, T.J. (1985). Monoclonal antibodies to osteoclastoma (giant cell bone tumours): definition of osteoclast specific cellular antigens. *Cancer Res.*, **45**, 5663.

HORTON, M.A. (1988). Osteoclast specific antigens. ISI Atlas of Science. *Immunology*, **1**, 35.

KELLY, P.M.A., BLISS, E., MORTON, J., BURNS, J. & MCGEE, J.O'D. (1988). Monoclonal antibody EBM/11: high cellular specificity for human macrophages. *J. Clin. Pathol.*, **41**, 510.

KNAPP, W., DOERKEN, K., GILKS, W.R. & 4 others (1989). (eds) *Leucocyte Typing IV*. Oxford University Press: Oxford.

KUKITA, T., MCMANUS, L.M., MILLER, M., CIVIN, C. & ROODMAN, G.D. (1989). Osteoclast-like cells formed in long-term human bone marrow cultures express a similar surface phenotype as authentic osteoclasts. *Lab. Invest.*, **60**, 532.

MUNDY, G.R., ALTMAN, A.J., GONDEK, M.D. & BANDELIN, J.G. (1977). Direct resorption of bone by human monocytes. *Science*, **196**, 1109.

MURRILLS, R.J., SHANE, E., LINDSAY, R. & DEMPSTER, D.W. (1989). Bone resorption by isolated human osteoclasts *in vitro*. *J. Bone Min. Res.*, **4**, 259.

PAPADIMITRIOU, J.M. & WALTERS, M.N.-I. (1979). Macrophage polykarya. *CRC Critical Rev. Toxicol.*, **6**, 211.

ROSAI, J. (1981). *Ackerman's Surgical Pathology*, Vol 2, 6th ed, p. 1380. C.V. Mosby: St. Louis.

TEITELBAUM, S.L., STERWART, C.C. & KAHN, A.J. (1979). Rodent peritoneal macrophages as bone resorbing cells. *Calcif. Tissue Int.*, **27**, 255.

WOOD, G.S., BECKSTEAD, J.H., TURNER, R.R., HENDRICKSON, M.R., KEMPSON, R.I. & WARNKE, R.A. (1986). Malignant fibrous histiocytoma tumor cells resemble fibroblasts. *Am. J. Surg. Pathol.*, **10**, 323.

WOOD, G.S., BECKSTEAD, J.H., MEDEIROS, L.J., KEMPSON, R.L. & WARNKE, R.A. (1988). The cells of giant cell tumor of tendon sheath resemble osteoclasts. *Am. J. Surg. Pathol.*, **12**, 444.